# A Case of Multiple Stun Attempts in a Bovine Due to Chronic Disease Process Causing Cranial Abnormalities

**DOI:** 10.3390/ani11010116

**Published:** 2021-01-08

**Authors:** Andrew Grist, Stephen B. Wotton

**Affiliations:** Bristol Veterinary School, University of Bristol, Bristol BS40 5DU, UK; steve.wotton@bristol.ac.uk

**Keywords:** animal welfare, abattoir, Animal Welfare Officer, captive bolt, cattle, restun, multiple stun attempts

## Abstract

**Simple Summary:**

Cattle that are to be processed for human consumption are routinely and legally rendered unconscious, to ensure that they cannot feel any pain, before the process of bleeding to cause brain death. The main method used in abattoirs is pneumatic or cartridge-powered captive bolt devices, which deliver a high velocity impact to the skull creating a severe concussion (stun). The penetrating captive bolts then continue travelling into the brain to prevent recovery from the stunned state by damaging the structures in the brain that are required for normal brain function. If this method does not work there is an obvious potential for the welfare of the animal to be compromised, so investigations are undertaken to establish the cause of any failures to reduce the risk of reoccurrence. This paper presents the results of such an investigation, where the cause of the failure of the device to stun was found to be due to the anatomy of the individual animal’s head. This unfortunate occurrence is rare but must be considered in any investigation of multiple stun attempts.

**Abstract:**

The preslaughter stunning of bovine animals is a legal requirement in the European Union, unless the animal is being slaughtered according to religious rite. The legislation also requires the investigation and review of stunning methods in cases of failure to stun. This paper presents the results of one investigation into the possible reasons for multiple stun attempts on an animal that received five shot applications. The head was hard frozen to prevent the deformation of brain structures during splitting along the sagittal plane, and then underwent macroscopic examination to assess the likely causes of the repeated stun attempts. In this case, a pre-existing chronic disease process produced anatomical variations of the cranial cavity, increasing the thickness of the sinuses of the frontal bone to a depth of 9 cm and filling the sinuses with a fibrinous pus deposit. It was therefore concluded that the anatomical variation produced by the chronic disease process, in addition to the energy absorption provided by the thicker hide and fibrinous pus, led to the failure of the stunning equipment to achieve the desired stunned state in the animal. As the animal displayed cranial variation before slaughter, a review of the stunning systems should include a requirement that animals displaying any abnormalities should be stunned with the highest-powered cartridge available or a free bullet.

## 1. Introduction

The preslaughter mechanical stunning of bovines has been undertaken in abattoirs in the United Kingdom (UK) since it was made a legal requirement in 1933 [1]. The main method used in UK premises, to achieve a state of brain dysfunction in bovines prior to exsanguination, is a penetrating captive bolt device [2]. Previous research was conducted, undertaking postmortem examinations of bovine heads that had received multiple shots (at least three attempts) during routine processing in abattoirs, to assess possible reasons for the multiple attempts. This work found that, in the small trial sample of 12 heads, 10 animals were reshot due to possible inaccuracies in position of the shot, one appeared to be due to gun or cartridge malfunction and one appeared to be most likely due to anatomical variation of the animal [3]. The European Union legislation [4] states that there should be monitoring systems in place for stunning, and it also requires a review of methods in case of failures to produce a stunned state.

It has long been established that the successful application of a captive bolt device produces a brain dysfunction (concussion) on impact with the cranium through differential acceleration of the brain in relation to the cranium and the subsequent propagation of pressure waves through the brain parenchyma disrupting brain function. [5,6,7,8,9]. The secondary action of the penetration of the cranium by the captive bolt is to attempt to prevent recovery from the stunned state by destroying vital brain structures such as the ascending activating system and the reticular activating system [6].

As part of the review of the method, in cases of failure of that method to produce a stunned state, external factors should be considered, such as gun performance [10], issues with the blank cartridges supplying the gas for the propulsion of the captive bolt [11,12], head movement and the positioning of the shot [13,14,15,16].

This paper presents the result of a postmortem examination of a bovine head that received five shots with penetrating captive bolts during routine processing in an abattoir.

## 2. Materials and Methods

The head from a Simmental heifer (25 months of age) was frozen to prevent distortion of the brain during sectioning and the external shot positions were noted. The head was then split along the sagittal plane to examine the wound tract and any macroscopic lesions encountered, using the methodology and equipment employed in the previous research into the use of macroscopic lesions as a tool to assess the effectiveness of penetrating captive bolt devices [3].

## 3. Results

**Figure 1:**
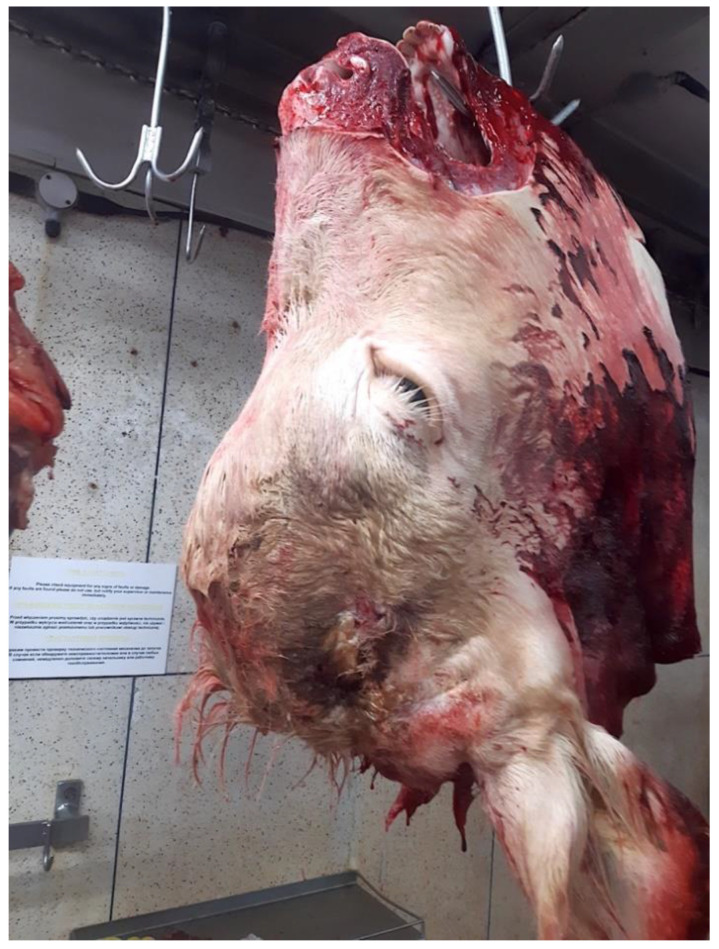
The head removed from carcass (Right Hand Side).

**Figure 2:**
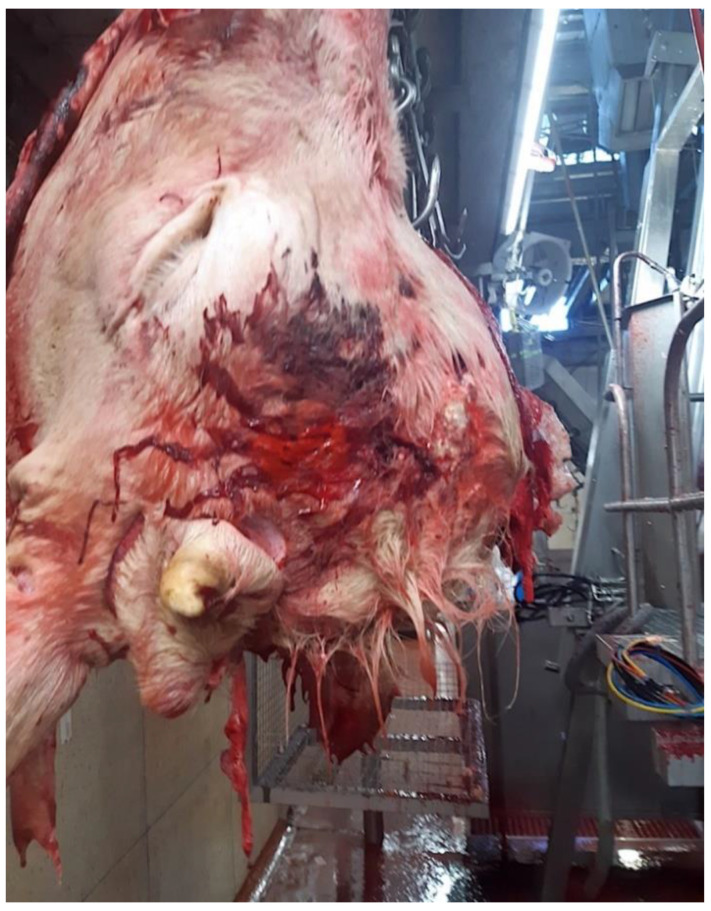
The head removed from carcass (Left Hand Side).

The photographs sent by the abattoir after the head was removed from the carcass (Figure 1 and Figure 2) show a prominent frontal bone, a small deformed horn on the left-hand side and no horn on the right. This suggests some form of malformation of the head or disruption during the growth period.

The penetration tracts of the applications were estimated using an 8 mm diameter trocar (Surgical Holdings UK, Essex, UK) to replicate the trajectory of the bolt (actual bolt diameter 11.71 mm). The shot numbering provided (with the exception of shot 5, which was reported) is used purely for descriptive purposes and does not indicate an assessment of the shot order.

### 3.1. Shot 1

**Figure 3:**
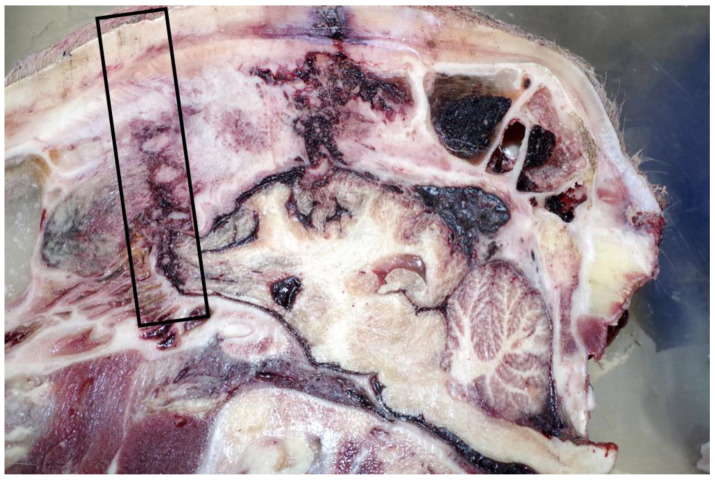
The trajectory of shot 1 (outlined), head right sagittal section.

Shot 1 was positioned and applied 5 cm rostral to the “ideal” position of the intersection of two imaginary lines drawn between the top of the eyes and the base of the opposite horn bud. The bolt trajectory (Figure 3) entered the frontal cranial vault, grazed the frontal lobe of the cerebrum and terminated in the fossa lobi piriformis, just rostral to the right olfactory recess of the lateral ventricle. This shot position and subsequent penetration tract would be considered inconsistent with the successful promotion of a state of concussion.

### 3.2. Shots 2–4

**Figure 4:**
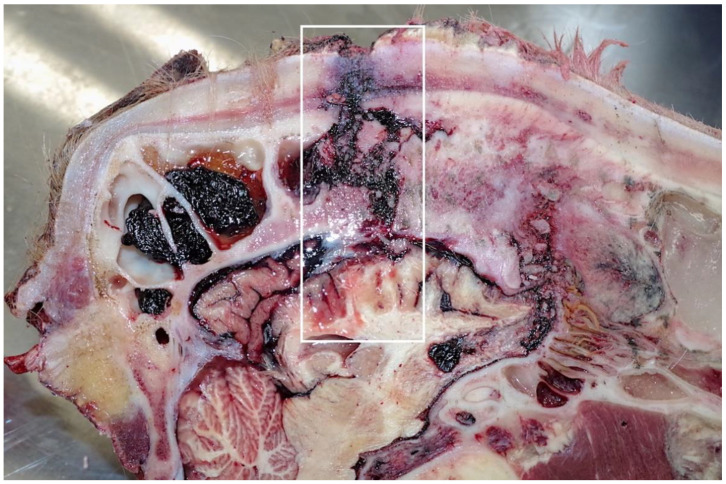
Shots 2–4. Left sagittal section.

These shots were placed in the “ideal” position although possibly too close to each other to have had the impact effect to stun. However, this is mitigated by the disease process affecting the skull structure. The tracts of the bolts (Figure 4) pass through the thickened hide (18 mm at the shot point) and enter the cranial vault, terminating at the parietal cerebrum, with petechial haemorrhages evident down to the level of the corpus callosum. The sinus thickness at this point was 9 cm.

### 3.3. Shot 5

**Figure 5:**
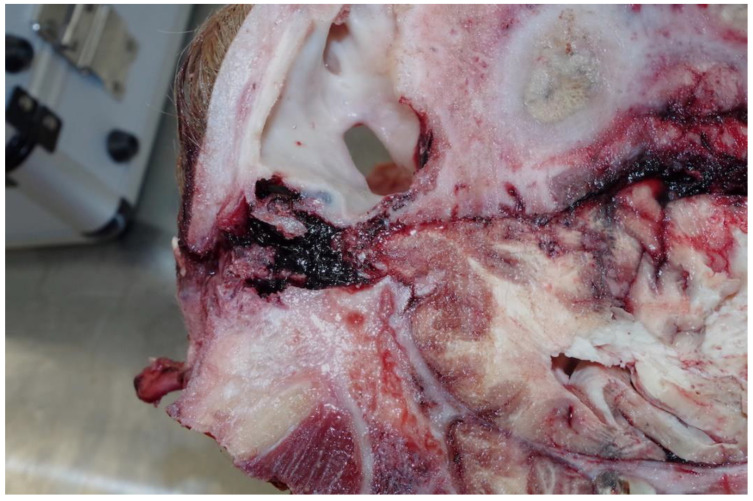
Shot 5 in poll position, left sagittal section.

Shot 5 was positioned and applied at the poll (the rear of head). The tract of the bolt passed above the cerebellum and into the occipital lobe of the cerebrum (Figure 5). Bone shards were evident in the tract, as was the traditional suctioning of brain material into the wound as the bolt retracted. This area is not considered suitable for stunning in England and Wales [16] as the level of unconsciousness has been shown to be light.

### 3.4. Disease Process

The abnormality of the head, suggested by the post shot photographs (Figure 1 and Figure 2) supplied by the abattoir, was confirmed when the head was sectioned.

**Figure 6:**
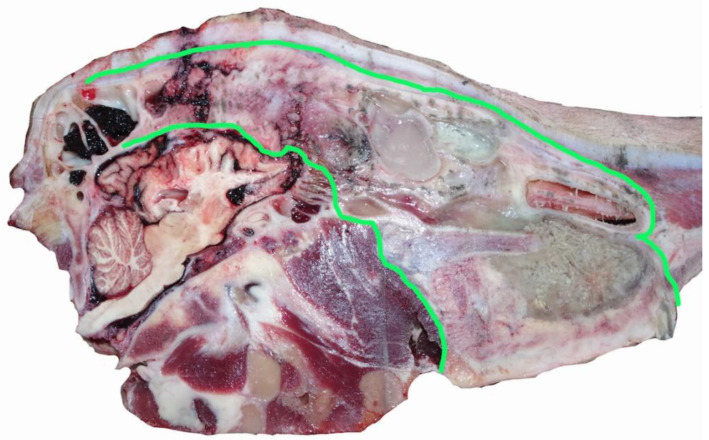
The area of the inflammatory process and chronic fibrous sinusitis with pus formation highlighted in green—left sagittal section.

**Figure 7:**
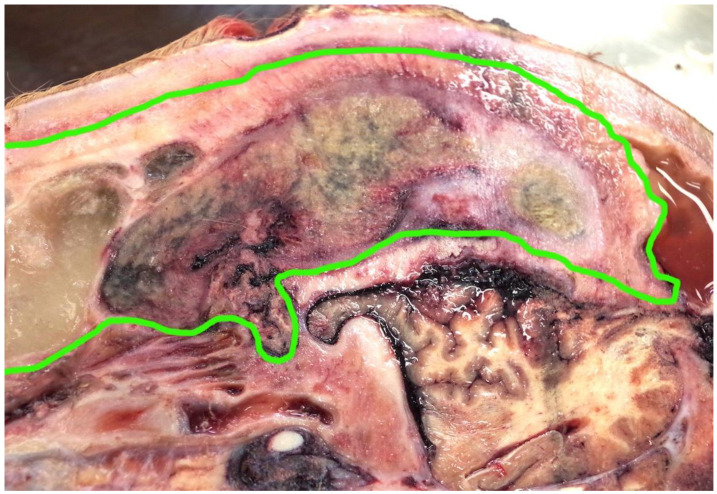
The right sagittal section showing chronic fibrous sinusitis with pus formation highlighted in green.

**Figure 8:**
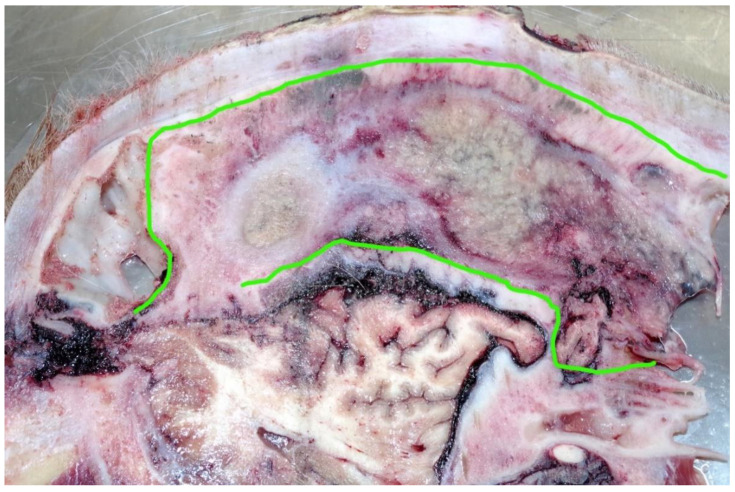
A head slice taken from 1.5 cm lateral to the midline. The left sagittal section showing chronic fibrous sinusitis with pus formation highlighted in green.

**Figure 9:**
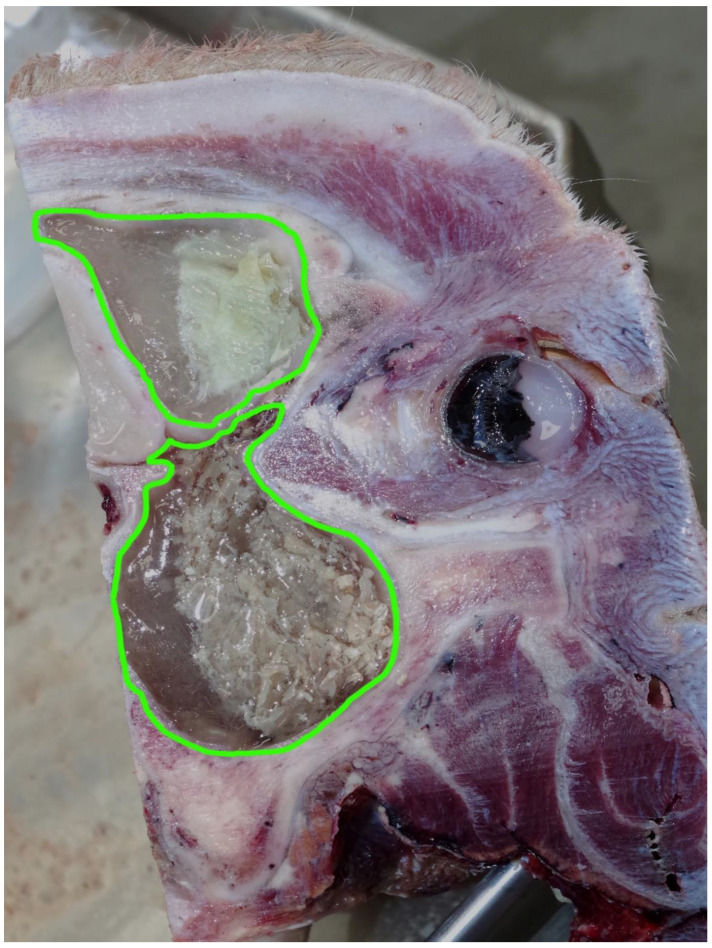
A frontal slice of the head (right side) through ocular orbit demonstrating chronic fibrous sinusitis with pus formation highlighted in green and inflammatory reaction apparent in the bone.

This animal had a fibrous sinusitis with a pus formation disease process which the evidence would suggest was chronic (Figure 6, Figure 7, Figure 8 and Figure 9). The differential diagnosis in this case includes chronic sinusitis, chronic *Actinomyces bovis* infection or possibly neoplastic formation.

## 4. Discussion

Before sectioning, the head presented with anomalies (one horn bud present and an enlarged frontal bone). One of the shots was placed lower than the “ideal” position, but this could have been an issue of landmarking with the head shape. The next three shots were accurately placed but were likely to have been ineffective due to the large mass of fibrous pus (a depth of 9 cm) plus thickened hide (18 mm) at the shot point, which would have absorbed the kinetic energy from the bolt. The final poll shot did not sever the spinal cord and entered the head dorsal to the cerebrum. In this case the poll shot is likely to have produced a level of concussion or brain damage that the others did not.

The postmortem examination demonstrated a pre-existing disease process of the sinuses extending into the cranial vault that would appear to be the contributing factor in the multiple stun requirement. Given the number of animals processed for human consumption, there will always be a small percentage of animals that present with anatomical variations. Although a useful guide, in addition to the behavioural indicators of the stunned state, examination of the external shot position does not provide the entire picture; splitting the head and examining the wound tracts of penetrating captive bolts can be more productive. Splitting the head along the sagittal plane demonstrated that the ineffectiveness of the stunning technique used was almost certainly due to anatomical variation in this one animal due to an existing disease process.

Although this examination, when combined with the results of the previous work [3] is a small-scale experiment, it nevertheless demonstrated that of 13 heads presented for postmortem examination due to receiving at least three stun attempts, 2 were due to anatomical variation. Given the number of animals stunned before slaughter [2] and the requirement of the European legislation to have standard operating procedures in place that include emergency procedures [1], these combined results should perhaps lead to a form of antemortem examination of cattle taking place by the abattoir staff to segregate any animal with an obvious deformity of the head from their standard stunning system and ensure that stronger cartridges or air pressure is used for these animals, or the option of a free bullet is available in these cases.

## 5. Conclusions

Previous work by the authors [3] raised the possibility that the macroscopic examination of cattle heads that receive multiple shots should be considered by abattoirs as part of the investigation, and a review of stunning procedures that should be carried out in the event of miss-stuns. Although the head presented demonstrated anomalies in external shape, the extent of the anomaly was only apparent when the head was split along the sagittal plane for examination. This work also suggests that it should be possible to segregate animals with cranial abnormalities prior to stunning, so that an alternative stunning method is used, or a procedure is preplanned for dealing with these animals to prevent the requirement for secondary stun attempts with the obvious animal welfare issues the initial failure to stun would elicit.
